# MRI findings prior to return to play as predictors of reinjury in professional athletes: a novel decision-making tool

**DOI:** 10.1186/s13244-022-01341-1

**Published:** 2022-12-27

**Authors:** Jaime Isern-Kebschull, Carles Pedret, Sandra Mechó, Ricard Pruna, Xavier Alomar, Xavier Yanguas, Xavier Valle, Ara Kassarjian, Javier Martínez, Xavier Tomas, Gil Rodas

**Affiliations:** 1 Department of Radiology, Hospital Clinic, University of Barcelona, C/Villarroel 170, 08036 Barcelona, Spain; 2 Clínica Diagonal, Barcelona, Spain; 3 Department of Radiology, Hospital de Barcelona, Barcelona, Spain; 4 FCBarcelona Medical Department, Barcelona, Spain; 5 Department of Radiology, Centres Mèdics Creu Blanca, Barcelona, Spain; 6 Musculoskeletal Radiology, Elite Sports Imaging, SL, Pozuelo de Alarcón, Madrid, Spain; 7 Medicine Sport Unit, Hospital Clínic-Sant Joan de Déu, Barcelona, Spain

**Keywords:** Magnetic resonance imaging, Return to play, Muscle injuries, Reinjury, Healing process

## Abstract

**Background:**

Because MRI has shown great accuracy in assessing acute muscle injuries, identification of risk factors for reinjury before return to play (RTP) in professional athletes during the healing process could be very relevant. We assessed the value of MRI findings prior to RTP as predictors of reinjury.

**Methods:**

Retrospective observational study of 59 professional athletes, mean age 26 years, with first-time acute muscle injury and successful rehabilitation ready to RTP. They underwent MRI within 6 days of the injury and within 7 days prior to RTP. The primary outcome was reinjury. Risk of reinjury was assessed using radiological signs in control MRI scans before RTP. The risk was classified as low, medium or high when none, one or two radiological signs were observed, respectively.

**Results:**

Reinjury occurred in 9 participants, with a rate of 15.2%. None of the baseline MRI-related variables was significantly associated with reinjury. In the control MRI scan performed within 7 days prior to RTP, three independent findings were significantly associated with reinjury. These included transversal and/or mixed connective tissue gap (*p* = 0.002), intermuscular oedema (*p* = 0.015) and callus gap (*p* = 0.046). In the predictive model of the risk of reinjury, the presence of two of these radiological signs, together with interstitial feathery oedema, was associated with a high risk of recurrence (OR 29.58, 95% CI 3.86–226.64; *p* = 0.001).

**Conclusions:**

In professional athletes with acute muscle injuries of the lower limbs successfully rehabilitated, some radiological signs on MRI performed shortly before RTP were associated with a high risk of reinjury.

**Supplementary Information:**

The online version contains supplementary material available at 10.1186/s13244-022-01341-1.

## Key points

- Certain radiological findings of acute muscle injury on MRI performed shortly prior to return to play may be considered risk factors of reinjury.
- There is a high risk of reinjury when two of these five radiological findings are present (connective tissue gap, loss of tendon tension, intermuscular oedema, callus gap and interstitial feather oedema).
- Despite appropriate progression during rehabilitation, MRI studies appear necessary in the decision to return to competition.

**Supplementary Information:**

The online version contains supplementary material available at 10.1186/s13244-022-01341-1.

## Background

Muscle injuries are a cause of considerable disability in professional athletes and result in a significant number of days lost annually during preseasons and seasons, as well as the economic costs that this entails for clubs and competitions [1, 2]. Tissue healing is a complex process and the goal of treatment is to facilitate a safe and timely return to play (RTP), particularly to minimise the risk of injury recurrence because of a premature or inappropriate RTP [3]. Reinjury is usually defined as an injury of the same type and location of the initial injury that occurs within a time limit of two months after the final rehabilitation day [4], whereas the definition of RTP includes criteria-based medical clearance and mental readiness for full training [5]. Moreover, RTP decisions should be made considering multiple factors, including recovery and rehabilitation.

MRI is a widely used imaging modality to assess and confirm the diagnosis of muscle injuries in elite athletes. Relevant muscle injury classification systems based on injury patterns characterised by MRI findings have been described [6–8]. MRI appearances of muscle injuries help to categorise the degree and help guide when the athlete may return to play [9–12]. A number of studies have associated MRI data obtained at the time of injury with recovery times [10–20], and other studies have explored the usefulness of MRI performed during the healing phase prior to RTP to anticipate recurrence of lesions [21–26], but definitive conclusions have not been drawn.

The aim of the study was to assess the value of MRI findings obtained at baseline (between day 1 and 6 after initial injury) and within 7 days prior to RTP as predictors of reinjury in professional athletes.

## Methods

### Design and participants

A retrospective study of all consecutive professional athletes from different sport disciplines of a professional club, who suffered from a first episode of acute muscle injury of the lower limbs over five consecutive seasons (2016–2021) during domestic and European cup matches was conducted. To be included in the study the following was required: to have available a diagnostic MRI study within 6 days after the acute injury, to have a good clinical progression during the rehabilitation period allowing to RTP and to have a control MRI scan within 7 days prior to RTP. Participants in whom surgery was the treatment of choice and accepted by the player (e.g. complete free tendon tear of the hamstrings) were excluded from the study. Athletes with low-grade mild injuries in whom a full rehabilitation program was not necessary and expected to allow return to play shortly were also excluded. Type of sports, muscles involved and salient clinical variables are included in Table 1.

**Table 1:** Sociodemographic and clinical variables

Variables	*N* (%)
Total	59 (100)
Type of sport	
Soccer	39 (66.1)
Futsal	15 (25.4)
Basketball	4 (6.8)
Rugby	1 (1.7)
Laterality	
Right	25 (42.4)
Left	34 (57.6)
Muscle	
Biceps Femoral	30 (50.8)
Rectus Femoris	9 (15.2)
Semimembranosus	7 (11.9)
Adductor Longus	3 (5.1)
Semitendinosus	3 (5.1)
Soleus	5 (8.5)
Pectineus	1 (1.7)
Medial gastrocnemius muscle	1 (1.7)
Anatomic location	
Tendinous	10 (17.0)
Myotendinous Junction with tendinous extension	32 (54.2)
Myotendinous Junction with muscular extension	1 (1.7)
Myofascial	10 (17.0)
Muscular	6 (10.2)

The study protocol was approved by the Institutional review board. Written informed consent was obtained from all participants.

### Medical RTP clearance protocol

During the recovery process, all participants underwent standardised rehabilitation for their injuries supervised by experienced sports physical therapists, based on the protocols previous described [27]. The protocol for treating muscle injuries is based on a specific multidisciplinary approach personalised to individual intrinsic conditions (sex, previous injuries, leg dominance, psychological factors and nutrition), extrinsic factors (playing level/position, time of the season workload and sport discipline), the mechanism of injury and location and characteristics of the injury demonstrated by MRI studies. Participants were clinically cleared for RTP if they were asymptomatic and had successfully completed the physiotherapy programme and sports-specific activities. The rehabilitation program also included monitorisation of biological parameters with GPS (e.g. physical and metabolic data) and progressive return to team training for short periods of time until the program has been finished successfully.

### MRI protocol

Players were imaged on Canon Medical Vantage Titan 3T MR scanner using dedicated surface coils. The MRI protocol for baseline assessment of muscle injuries consisted of 5 sequences: 3 fluid-sensitive sequences (intermediate-weighted) fat suppressed with an intermediate-low echo time (TE) in the 3 planes, and 2 T1-weighted sequences in axial and coronal planes. The MRI protocol for the control assessment of muscle injury healing before RTP consisted of 3 sequences: 2 intermediate-weighted fat suppressed in coronal and axial planes and 1 T1-weighted sequence in axial plane. Details of the MRI protocol are provided in Additional file 1: appendix 1.

### MRI assessments and data collection

MRI images obtained were reviewed independently by three experienced musculoskeletal radiologists with at least 5 years of experience who were blinded to the clinical history and physiotherapy progression of athletes. Any discordant results were resolved by consensus. In all cases, MRI studies were evaluated using a systematic approach [28] as follows: (a) anatomical assessment on T1-weighted sequences (axial and coronal): individual muscular anatomy, anatomical variants, residual changes from previous lesions (scarring, atrophy) and vascular structures; and (b) lesion assessment on T1 and T2/fluid sensitive sequences (acute lesions on T2-weighted sequences and evidence of scarring on both T1- and T2-weighted sequences): location of the lesion (proximal, middle, distal), anatomical structures involved (tendon, aponeurosis, fascia, muscle fibres) and pattern of the tear, oedema and/or scar (in case of MRI prior to RTP).

The evaluation of baseline MRI images included the following variables: connective tissue (tendon, raphe, septa and aponeurosis) tear/disruption, muscle fibres tear/disruption, loss of tendon tension (wavy sign), loss of pennation angle, oedema and previous scar. Images obtained in control MRI scans prior to RTP were compared with baseline MRI scans in order to evaluate the repair process focused on the restitution of tissue architecture and callus formation and included the following variables: connective tissue and muscle fibres gap, scar/callus gap, loss of tendon tension, loss of pennation angle, oedema, scar maturation and scar morphology. The definition of MRI terms and categorisation of variables is shown in Table 2.

**Table 2:** MRI definition of terms and categorisation of variables

Variables	Evaluation	Category	MRI findings
Connective tissue tear (tendon, raphe, septa and aponeurosis)	Orientation of the disruption respect to the longitudinal axis of the tendon	Absent Transversal Longitudinal Mixed	Hyperintense gap on T2-weighted images with clear loss of connective tissue continuity
Loss of tendon tension (wavy sign)	Location of irregularity of the tendon	Absent Present	Loss of longitudinal alignment in the tendon in the form of an undulating (wavy) pattern
Muscle fibres tear	Presence of a measurable gap	Absent Present	Hyperintense gap on T2 with clear loss of muscle fibres continuity Isolated (between fibres) or in the myoconnective interface, and at this last level associated with gaps of connective tissue
Loss of pennation angle	Anchorage of muscle fibres	Absent Present	Loss of anchorage of the muscular fibres with the connective tissue
Oedema Intramuscular	High signal intensity within the muscle on fluid sensitive sequences	Adaptive (cotton-like pattern) Feathery oedema (dissecting the Interstitial space between muscle fibres)	Muscle area of low to intermediate signal intensity on T1-weighted images and high signal intensity on fluid sensitive sequences Feathery pattern: oedema that dissects the interstitial space between muscle fibres Cotton-like pattern: more diffuse and poorly defined without architectural muscle distortion
Oedema Intermuscular	Intermuscular fluid on fluid sensitive sequences	Absent Present	Presence of fluid in the intermuscular space
Improvement in oedema	Total or partial radiological regression of intramuscular and/or intermuscular oedema	Yes No	Decrease in the extent of the both forms of oedema in control MRI prior to RTP as compared with baseline MRI
Scar tissue	Morphology of scar tissue	Hypertrophic Elongated Round Fusiform	Thickness greater than myoconnective environment (hypertrophic) The morphology of the callus is given by its thickening in the longitudinal plane (fusiform), in the axial plane (rounded) or uniform with respect to the intact tendon (elongated)
	Maturation of scar tissue	Immature Mature	A T2 hyperintense centre surrounded by a very thin hypointense peripheral line (immature), progressive filling in and losing signal Formation of hard callus with low signal in both T1- and T2-weighted images (mature)
	Callus gap (disruption)	Absent Present	Persistence or appearance of hyperintense gap on T2-weigthed images with clear transversal loss of its continuity

Risk of reinjury was defined in the presence in control MRI scans prior to RTP of typical radiological signs of acute muscle injury, which have been reported to be associated with prolonged RTP [10–20]. These signs included the persistence or appearance of transversal and/or mixed (transversal and longitudinal discontinuity) connective tissue gap, intermuscular oedema, persistence or progression of feathery oedema to which persistence or appearance of a callus gap was added. The risk of reinjury was classified as low, medium or high when none, one or two of these radiological signs were observed, respectively.

In all participants, the following clinical variables were recorded: age, sport, leg dominance, muscle affected, type of lesion, MRI findings at baseline and prior RTP, and time interval between RTP and reinjury.

### Outcome

**Fig. 1:**
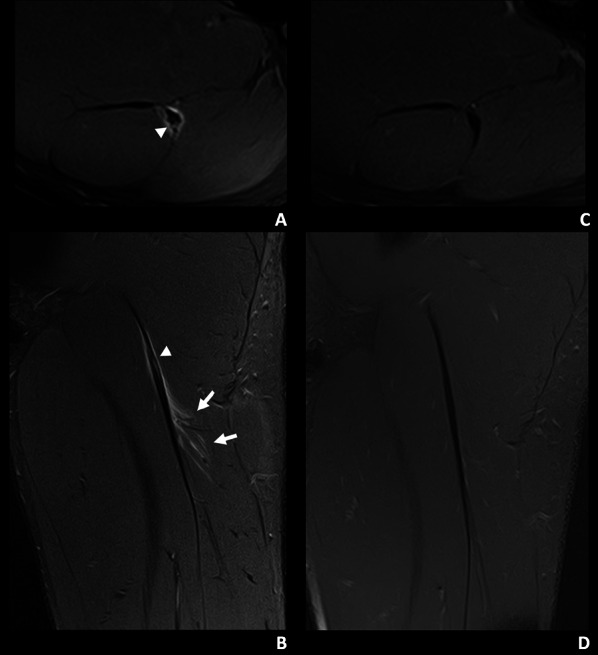
A 19-year-old left leg dominant football player of the non-reinjured group with mild tenderness months ago in the proximal portion of the left thigh. During a sprint in a match he experienced sudden pain in the proximal hamstrings region. **A**, **B** Axial and coronal T2-weighted fat-saturated images from a baseline MRI show a myotendinous injury of the proximal MTJ of the biceps femoris long head, I Pp 3r 0 (Muscle Location Grade and Reinjury [MLG-R] classification [33]). There is a mixed intratendinous tear (arrowhead) with peritendinous oedema and trace interstitial oedema (arrows). **C**, **D** Axial and coronal T2-weighted fat-saturated images from a control MRI, 48 days later, show a mature tendinous scar without oedema

Reinjury after RTP was the primary outcome of the study. The diagnosis of reinjury was established by the reappearance of symptoms on returning to sport competition activities and confirmed by MRI (reinjury group). The time interval that was considered for reinjury after RTP was 2 months. Players who resumed competition normally were included in the non-reinjury group (Fig. 1).

### Statistical methods

Categorical variables are expressed as frequencies and percentage, and continuous variables as median and range. The distribution of MRI-related variables in reinjury and non-reinjury groups was compared using the Fisher’s exact test. Statistical significance was set at *p* = 0.01 (two-sided). Variables independently associated with reinjury after RTP were analysed in a logistic regression model. The results are expressed as odds ratio (OR) and 95% confidence interval (CI). Statistical analyses were performed using Stata 14 SE for Windows (StataCorp, College Station, TX, USA).

## Results

### Clinical characteristics, location and injury type and RTP

**Fig. 2:**
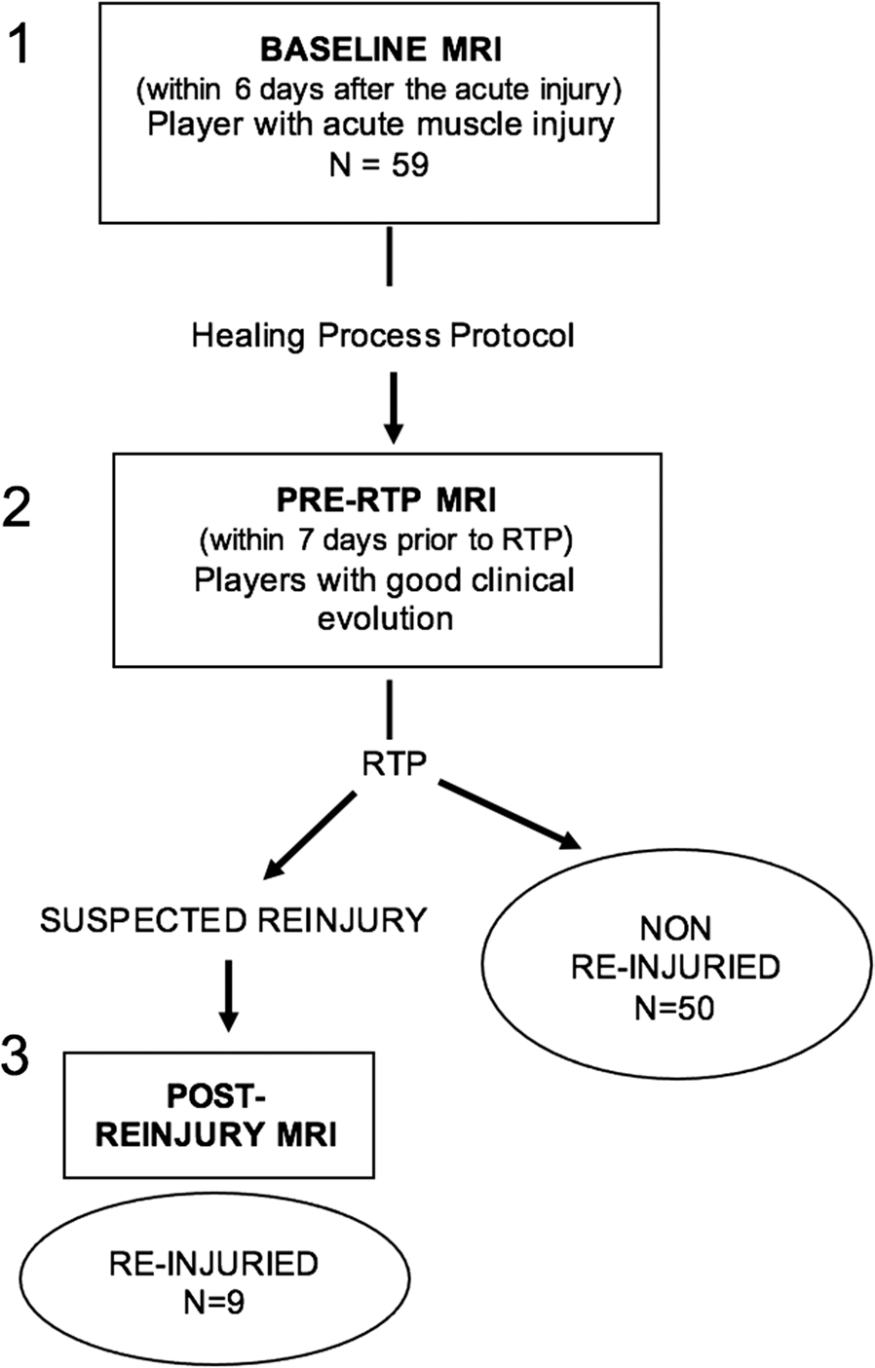
Flow diagram of patients with muscle injuries of the lower extremities included in the study

During the study period, a total of 69 male professional athletes were eligible to participate in the study but 10 were excluded for the following reasons: indication of surgical treatment (*n* = 5) and current index lesion as a reinjury of a previous injury (*n* = 5). The flowchart of the study population is shown in Fig. 2. Therefore, the study population included 59 athletes, with a mean age of 26 years (range 22–30 years). The baseline MRI examination was performed at 24 h after injury in 45 participants (76.3%), after 2 days in 6 (10.2%), after 3 days in 4 (6.8%) and after 4 days in the remaining 4 (6.8%).

The median RTP was 35 days (range 8–68). Duration of the median RTP varied according to the injured muscle group, with 43 days for adductor longus, 39 days for pectineus, 38 days for biceps femoris, 36 days for soleus, 35 days for medial gastrocnemius, 30 days for rectus femoris, 26 days for semimembranosus and 24 days for semitendinosus. In relation to the injured muscular structure, median RTP was 40 days for myotendinous junction (MTJ) with tendinous extension, 35 days for tendinous lesions, 29 days for muscular injuries and 23 days for myofascial tears.

### Reinjury and MRI findings

The distribution of variables recorded in baseline MRI studies in the non-injury and reinjury groups is shown in Table 3. Overall findings were consistent with typical MRI characteristics of acute muscle injuries, including involvement of the thick connective tissue of the mixed tendon tear type, muscle fibre tear at myoconnective junction (MCJ) and loss of tendon tension and muscle pennation angle. Also, intermuscular oedema and interstitial feather pattern oedema were common findings.

**Table 3:** Differences in baseline MRI findings between professional athletes with and without reinjury

Variables	Non-reinjury (*n* = 50)	Reinjury (*n* = 9)	OR (95% CI)	*p* value
Connective tissue rupture				
Absent	14 (28.0)	1 (11.1)		
Transversal	1 (2.0)	1 (11.1)		
Longitudinal	3 (6.0)	1 (11.1)		
Mixed	32 (64.0)	6 (66.8)	1.19 (0.66–2.17)	0.564
Loss of tendon tension				
Absent	16 (32.0)	1 (11.1)		
Present	34 (68.0)	8 (88.9)	3.76 (0.43–32.71)	0.229
Muscle fibres tear				
Absent	7 (14.0)	1 (11.1)		
Present	43 (86.0)	8 (88.9)	1.30 (0.14–12.08)	0.816
Loss of pennation angle				
Absent	7 (14.0)	0	NC	
Present	43 (86.0)	9 (100.0)		
Adaptive oedema				
Absent	48 (96.0)	9 (100.0)	NC	
Present	2 (4.0)	0		
Feather oedema				
Absent	6 (12.0)	0	NC	
Present	44 (88.0)	9 (100.0)		
Oedema intermuscular				
Absent	14 (28.0)	9 (100.0)	NC	
Present	36 (72.0)	0		
Previous scar				
Absent	27 (54.0)	4 (44.4)	1.46 (0.62–3.47)	0.389
Subacute	14 (28.0)	2 (22.2)		
Chronic	9 (18.0)	3 (33.3)		
Previous scar MCJ location				
Absent	27 (54.0)	4 (44.4)	1.23 (0.47–3.27)	0.672
Same	19 (38.0)	4 (44.4)		
Different	3 (6.0)	1 (11.1)		
Contralateral	1 (2.0)	0		
Previous scar third involved leg				
Same	45 (90.0)	7 (77.8)	1.54 (0.65–3.65)	0.331
Distal	1 (2.0)	0		
Proximal	3 (6.0)	2 (22.2)		
Contralateral	1 (2.0)	0		

*NC* not calculated, *MCJ* myoconnective junction

In relation to the primary outcome of the study, the reinjury rate was 15.2% (9/59). The median time elapsed between MRI prior RTP and reinjury was 9 days (range 0–32). The biceps femoris (long head) was the most commonly reinjured muscle as seen in 5 cases (55.5%) followed by the rectus femoris, semimembranosus, semitendinosus and soleus in 1 (11.1%) case each. Tissues involved were MTJ with tendinous extension in 7 cases, followed by tendinous and myofascial tear in 1 case each.

In the control MRI scan performed within 7 days prior to RTP (Table 4), three independent findings were significantly associated with reinjury. These included transversal and/or mixed connective tissue gap (*p* = 0.002), intermuscular oedema (*p* = 0.015) and callus gap (*p* = 0.046). In the predictive model of the risk of reinjury, the presence of two of the following radiological signs: transversal and/or mixed connective tissue gap, loss of tendon tension, intermuscular oedema, callus gap and interstitial feather pattern oedema, was associated with a high risk of recurrence (OR 29.58, 95% CI 3.86–226.64; *p* = 0.001) (Table 4).

**Table 4:** Differences in control MRI findings (within 7 days prior to return to play) between professional athletes with and without reinjury

Variables	Non-reinjury (*n* = 50)	Reinjury (*n* = 9)	OR (95% CI)	*p* value
Transversal and/or mixed connective tissue gap				
Absent	49 (9.8)	5 (55.6)	39.2 (3.64–422.14)	0.002
Present*	1 (2.0)	4 (44.4)		
Loss of tendon tension				
Absent	42 (84.0)	6 (66.8)	2.63 (0.54–12.7)	0.231
Present*	8 (16.0)	3 (33.3)		
Longitudinal connective tissue gap				
Absent	44 (88.0)	7 (77.8)	2.10 (0.35–12.52)	0.417
Present	6 (12.0)	2 (22.2)		
Muscle fibres tear				
Absent	43 (86.0)	7 (77.8)	1.76 (0.30–10.23)	0.532
Present	7 (14.0)	2 (22.2)		
Loss of pennation angle				
Absent	38 (76.0)	7 (77.8)	1	
Present	12 (24.0)	2 (22.2)	0.90 (0.17–4.95)	0.908
Global improvement in oedema				
Absent	21 (42.0)	4 (44.4)	0.91 (0.22–3.78)	0.891
Present	29 (58.0)	5 (55.6)		
Adaptive oedema				
Absent	5 (10.0)	2 (22.2)	0.39 (0.06–2.41)	0.310
Present	45 (90.0)	7 (77.8)		
Feather oedema				
Absent	30 (60.0)	4 (44.4)	1.87 (0.45–7.85)	0.389
Present*	20 (40.0)	5 (55.6)		
Intermuscular oedema				
Absent	35 (70.0)	2 (22.2)	8.17 (1.52–43.99)	0.015
Present*	15 (30.0)	7 (77.8)		
Scar maturity				
Immature	18 (36.0)	5 (55.6)	0.45 (1.11–1.89)	0.276
Mature	32 (64.0)	4 (44.4)		
Hypertrophic scar				
Absent	13 (26.0)	2 (22.2)	1.09 (0.23–5.10)	0.916
Present	36 (72.0)	7 (77.9)		
Scar morphology				
Absent	10 (20.0)	2 (22.2)	1.15 (0.39–3-35)	0.799
Elongated	34 (68.0)	6 (66.8)		
Round	5 (10.0)	0		
Fusiform	1 (2.0)	1 (11.1)		
Callus gap				
Absent	46 (92.0)	6 (66.7)	5.75 (1.03–32.17)	0.046
Present*	4 (8.0)	3 (33.3)		
Risk of reinjury				
Low: absence of any radiological sign	38 (76.0)	1 (11.11)		
Medium: presence one radiological sign	12 (24.0)	2 (22.22)		
High: presence of two radiological signs	0 (0.0)	6 (66.67)	29.58 (3.86–226.64)	0.001

*Radiological signs included in the model of the risk of reinjury

## Discussion

The present findings show that certain radiological signs in MRI studies performed within 7 days prior to RTP in professional athletes after a first episode of muscle injury and who have been clinically cleared for RTP are useful as predictors of reinjury. Tendon tear and loss of tendon tension, findings seen in MRI studies shortly after acute muscle injuries and which have previously been reported to be associated with prolonged recovery times [10–20], as well as a callus gap were predictors of reinjury, especially when two or more signs were present. In our study, the injuries that were associated with a greater RTP were those that affected the myotendinous junction with tendon extension, followed by pure tendon injuries. Moreover, the most frequently injured and re-injured muscle was the biceps femoris, also showing the greatest range of RTP (17–68 days) given the complexity of the histoarchitecture of the connective tissue.

Data of MRI at baseline were not useful in predicting the risk of reinjury, a finding which is consistent with prior studies [29, 30]. It seems that if the recovery process is adequate and fully adapted to specific conditions of the lesion, reinjury could not be anticipated according to baseline MRI data.

To our knowledge, assessment of predictive imaging variables in control MRI shortly before RTP using logistic regression analysis has not been previously documented. The present results are clinically relevant, not only because of the identification of specific predictive radiological signs per se, but also for emphasising the role of MRI performed prior to RTP, when from a clinical point of view the player is asymptomatic and ready to return to competition.

**Fig. 3:**
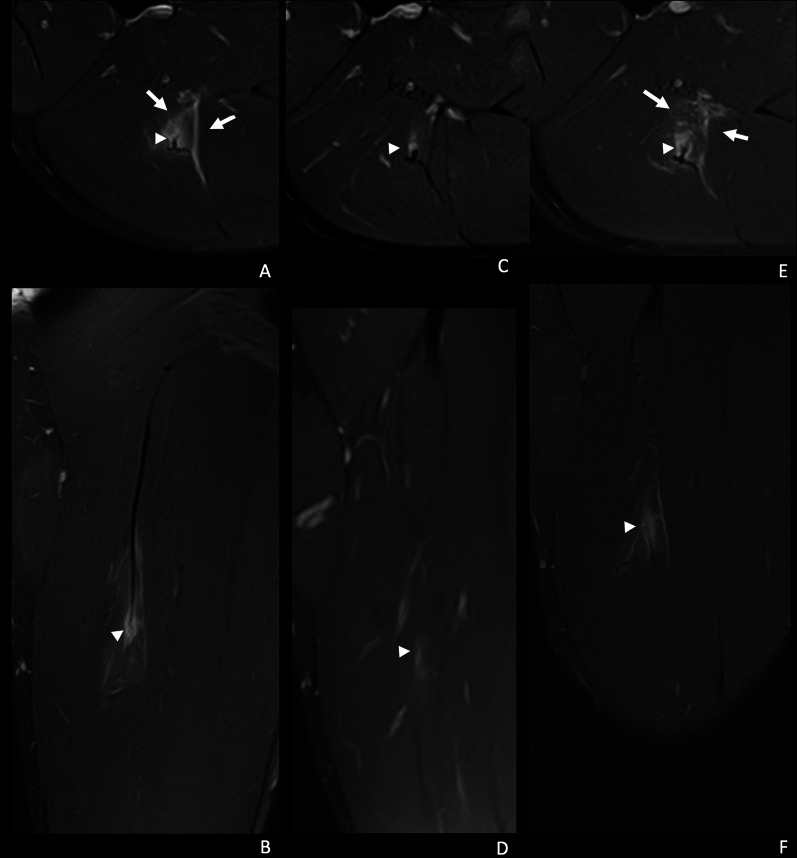
A 21-year-old right leg dominant football player. He presented during a training session, with tenderness in the middle third of the posterior aspect of the left thigh, and inability to continue training. **A**, **B** Axial and coronal T2-weighted fat-saturated images from a baseline MRI show myotendinous injury of the intramuscular portion of the proximal MTJ of the biceps femoris long head, I Mp 3r 0 (MLG-R classification [33]). There is a transversal tear of the connective tissue with loss of tension (arrowhead) and interstitial and intermuscular oedema (arrows). **C**, **D** Axial and coronal T2-weighted fat-saturated images of the pre-RTP MR (37 days post-injury), show a small transversal peripheral tendinous tear (arrowhead) with a trace interstitial oedema. **E**, **F** Reinjury 1 day later. Axial and coronal T2-weighted fat-saturated images show a thicker transversal tendinous tear with fibre muscle tear (arrowhead) and more interstitial oedema and intermuscular oedema (arrows)

The appearance or persistence of a transversal gap in the tendon, regardless of its loss of tension, was an independent predictor of reinjury (Fig. 3). Previous studies have consistently reported that compromise due to tendon rupture is related to a worse prognosis and longer time to recovery [18, 19, 22, 31]. Loss of tendon tension may appear when a significant cross-sectional area of a tendon is injured, which manifests on imaging as a wavy tendon. However, if a tendon is degenerated or thickened due to prior injuries, this loss of tendon tension may not be readily apparent [28]. In our study, loss of tendon tension was not associated with reinjury. A recent study of Vermeulen et al. [23] of hamstring intramuscular tendon injuries in 41 athletes showed partial- or complete thickness tendon discontinuities at RTP downplaying the importance of visualising this imaging sign. In some cases of intramuscular tendon injuries, the persistence of disruption of the tendon with partial longitudinal extension but without associating reinjuries was observed [23]. Of note, three differentiating factors of our study as compared with that of Vermeulen et al. [23] include the following: (1) in our population, all muscle tendon and myoconnective injuries of multiple muscle groups were included, (2) the criteria used to define discontinuity were different, in our study defined as the presence of the presence of a new or persistent measurable gap of the thick connective tissue (including tendon) defined as gap of hyperintense signal in fluid-sensitive sequences, and (3) the interpretation and categorisation of the variables of the MRI performed prior to RTP were compared with findings of the baseline MRI.

The visualisation or persistence of isolated tear of muscle fibres, tears at the myofascial junction and the loss of pennation angle of the muscle fibres at control MRI prior to RTP were not related to reinjury. In our opinion, and as previously described [28, 32], the thickness of the connective tissue decreases as you get further from the muscle origin. As such, at the level of the myofascial unit, there are no consistent MRI signs to indicate the degree of involvement of the connective tissue, except for the presence of intermuscular and/or interstitial oedema.

Changes such as oedema are common indirect findings of a muscle injury on MRI at RTP in athletes who are clinically recovered from an acute hamstring injury [13, 21, 33]. However, incomplete resolution of oedema does not seem to prevent successful RTP [12]. Also, global improvement in oedema was frequently observed in non-reinjury and reinjury groups in similar percentage. In other studies, different patterns of muscle interstitial oedema in both baseline and control MRI prior to RTP have not been assessed, with previous results primarily based on the measurable extent of oedema [20, 23, 26]. The cotton-like oedema that we have shown in our study is similar to the peritendinous ovoid or subfascial ring oedema indicated by Kho et al. [34] in professional soccer players with suspected acute thigh muscle injuries, that they called exercise-related signal. We found that interstitial oedema in a feathery pattern when seen together with certain other signs (transversal and/or mixed connective tissue gap, loss of tendon tension, intermuscular oedema, callus gap) was a predictor of reinjury. The presence of isolated intramuscular oedema was not associated with reinjury. The assessment of intramuscular oedema is complex and the different patterns of cotton-like (adaptive), interstitial feathery oedema and intermuscular fluid on fluid sensitive sequences can be observed at baseline and control MRI studies, although cotton-like oedema was only seen in 4% of participants at baseline, increasing to 40% and 55.6% in non-reinjury and reinjury groups, respectively, at MRI prior to RTP as a normal sign of adaptation during the healing process.

**Fig. 4:**
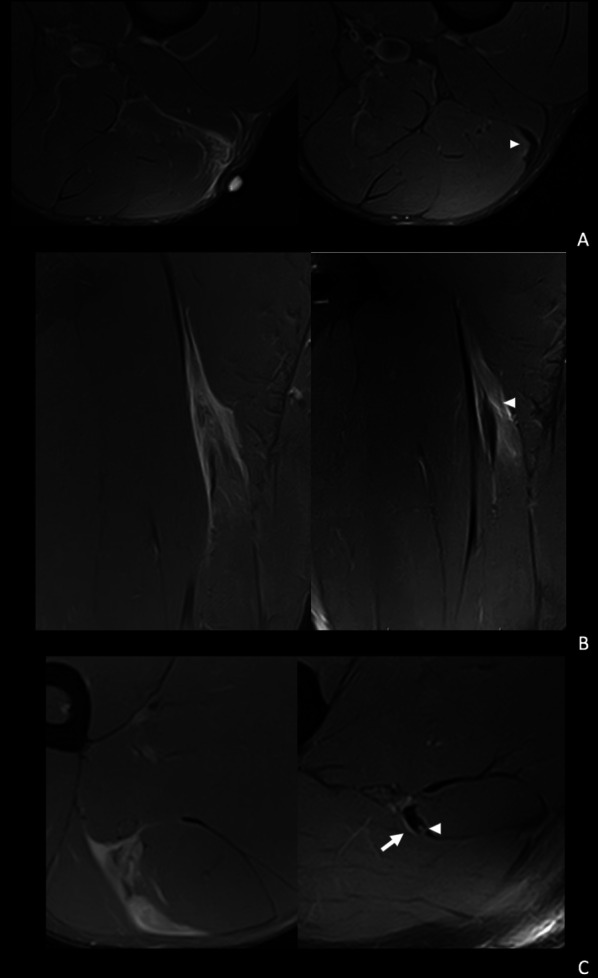
Spectrum of findings related to risk of reinjury. **A** A 31-year-old right leg dominant futsal player. Axial T2-weighted fat-saturated images shows: from a baseline MRI (left) a distal myotendinous injury of the left biceps femoris long head, I D_d_ 3^r^ 0 (left). In the pre-RTP image (right) we can see a mature scar and a mild adaptive oedema (arrowhead). No signs associated with a higher reinjury risk. **B** 21-year-old right leg dominant football player. Coronal T2-weighted fat-saturated images from a baseline MRI show a proximal myotendinous injury of the left biceps femoris long head, I P_p_ 3^r^ 0 (left). In the pre-RTP image (right) we can see a mature hypertrophic scar and interstitial oedema (arrowhead). This was considered medium risk for reinjury. **C** 22-year-old left leg dominant football player. Axial T2-weighted fat-saturated images from a baseline MRI (left) show a proximal myotendinous injury of the left biceps femoris long head, I P_p_ 3^r^ 1. In the pre-RTP axial T2 image (right) we can see proximal to the previous injury tendinous tear (arrowhead) and intermuscular oedema (arrow). This was considered high risk for reinjury

The formation of scar tissue has been described as a progressive process beginning as a soft callus on the 8th day post-injury, which shows a T2 hyperintense centre surrounded by a very thin hypointense peripheral line, progressively filling in and losing signal, until forming a hard callus with low signal in both T1 and T2-weighted images [25, 28, 32]. In the present study, the degree of maturity, hypertrophy or morphology of the callus were not related to reinjury. However, persistence or appearance of a transverse tear in the form of a measurable gap in the control MRI prior to RTP was a significantly predictive radiological sign of reinjury (Fig. 4). It is important to become familiarised with the evolutionary stages of the callus over time, since in some cases there may be irregularities of the scar (notchy callus), which suggests adaptive changes, which are not seen as a complete gap, and may not necessarily be considered a tear. The presence of a transversal gap in a clinically asymptomatic patient after having completed an adequate physiotherapy and rehabilitation process could be due to a delay in the complete formation of the callus or to overload, both of which may limit incorporation of the player to high level training.

The limited number of reinjuries observed over five seasons could be viewed as a limitation of the study, but our rate of 15.2% is consistent with 16–18% reported in epidemiological studies [1, 2]. It is possible that small number of reinjuries could have resulted in certain predictors of reinjury based on pathophysiological reasons, such as the degree of maturity of the callus, not reaching statistical significance in the current study. Further studies with a larger number of subjects and reinjury events are needed to extrapolate the present findings to professional female athletes, other sports, and other muscle groups. Although all participants underwent standardised physical therapy protocols according to the type of acute injury, differences according to the sport were not evaluated. The present findings encompassed a few numbers of each muscle, so that it would be interesting to assess the value of MRI findings prior to RTP focused on potential predictive signs for each individual muscle. A study including a variety of muscle groups would be also interesting to assess significant differences in susceptibility of recurrence. On the other hand, the potential influence of the athletes’ age upon the time to return to sport was not analysed. Despite these limitations and the retrospective design, MRI scans were independently and blindly evaluated by three expert musculoskeletal radiologists following a systematic and protocolised approach, the steps of which have been previously described [28] (Additional file 1). It is also important to consider that the concept of RTP is multifactorial and in the context of different factors related to the player, a good clinical progression during the rehabilitation period was an inclusion criterion and all control MRI studies were performed within 7 days prior to RTP.

Despite the fact that in recent years certain publications have downplayed or questioned the role of MRI in monitoring muscle injuries [21, 26], the current results demonstrate that certain MRI findings are predictive of reinjuries and may be a useful tool in the multifactorial decision-making process of recovery and RTP [35–37].

## Conclusion

In this pilot study of professional athletes with first-time acute muscle injuries of the lower limbs with successful rehabilitation, specific radiological signs detected on control MRI studies performed shortly prior to RTP were associated with a high risk of reinjury. These included transversal and/or mixed connective tissue gap, loss of tendon tension, intermuscular oedema, callus gap and interstitial feather oedema pattern, with high risk for reinjury when two of these findings, were present.

## Supplementary Information

**Additional file 1.** Appendix 1.

## Data Availability

The datasets used and/or analysed during the current study are available from the corresponding author on reasonable request.

## Abbreviations

MCJ: Myoconnective junction
MRI: Magnetic resonance imaging
RTP: Return to play
TE: Echo time
