# Supplementary material for: MRI findings prior to return to play as predictors of reinjury in professional athletes: a novel decision-making tool
Source: Insights Imaging. 2022 Dec 27;13:203. doi: 10.1186/s13244-022-01341-1 (PMC9794673; doi:10.1186/s13244-022-01341-1)
Supplement: Supplementary file 1 — Additional file 1. Appendix 1. [file 13244_2022_1341_MOESM1_ESM.pdf]

ELECTRONIC SUPPLEMENTARY MATERIAL

MRI findings prior to return to play as predictors of reinjury in professional athletes: a novel decision-making tool

Appendix 1

MRI protocol

The subject was positioned in supine decubitus and examination of the injured limb around the area marked with the skin vitamin marker (according to clinical findings) was exclusively performed. The MRI protocol for baseline assessment of muscle injuries consisted of 5 sequences: 3 fluid-sensitive sequences (intermediate-weighted) fat suppressed with an intermediate echo time (TE) in the 3 spatial planes: coronal (repetition time [TR]/TE 5000/44 ms, section thickness 2.5 mm, interslice thickness gap 0.6 mm, field of view [FOV] 26x29 mm^2, slice thickness 2.5 mm, matrix 288×320); axial (TR/TE 5200/44 ms, interslice thickness gap 0 mm, FOV 25x25 mm^2, slice thickness 3.5 mm, matrix 256×256); and sagittal (TR/TE 3700/60 ms, interslice thickness gap 0 mm; FOV 27x24 mm^2, slice thickness 2.8 mm, matrix 192x272) and 2 T1-weighted sequences in axial (TR/TE 900/11 ms; interslice thickness gap 0 mm, FOV 25x25 mm^2, slice thickness 3.5 mm, matrix 352x352) and coronal (TR/TE 980/11 ms, interslice thickness gap 0.6 mm, FOV 26x29 mm^2, slice thickness 2.5 mm, matrix 288x320) planes. The MRI protocol for the control assessment of muscle injury healing before return to play (RTP) consisted of 3 sequences: 2 intermediate-weighted fat suppressed in coronal and axial planes, and 1 T1-weighted sequence in axial plane with the same technical parameters.

Appendix Table 1

MLG-R Classification (FCB Barcelona and Aspetar Classification)[35,36,37]

FCB Barcelona and Aspetar Classification	
Mechanism of injury (M)	I (indirect)<br>T (direct)
Locations of injury (L)	P (proximal)<br>M (middle)<br>D (distal)
Grading of severity (G)	0 (Negative MRI)

	1 (Oedema without intramuscular hemorrhage or architectural distortion)
	2 (Oedema with minor muscle fiber architectural distortion ± minor intermuscular hemorrhage, but no gap between fibers)
	3 (Any quantifiable gap between fibers with partial retraction ± intermuscular hemorrhage)
Tendinous lesion (r)	r (presence or not depends of tendinous involvement)
No. of muscle re-injuries (R)	0: 1st episode<br>1: 1st re- injury<br>2: 2nd re- injury...
